# New horizons in evidence synthesis for older adults

**DOI:** 10.1093/ageing/afad211

**Published:** 2023-11-13

**Authors:** Nicola Cooper, Evi Germeni, Suzanne C Freeman, Nishant Jaiswal, Clareece R Nevill, Alex J Sutton, Martin Taylor-Rowan, Terence J Quinn

**Affiliations:** NIHR Evidence Synthesis Group @Complex Review Support Unit; Department of Population Health Sciences, University of Leicester, Leicester, UK; NIHR Evidence Synthesis Group @Complex Review Support Unit; Health Economics and Health Technology Assessment (HEHTA), School of Health and Wellbeing, University of Glasgow, Glasgow, UK; NIHR Evidence Synthesis Group @Complex Review Support Unit; Department of Population Health Sciences, University of Leicester, Leicester, UK; NIHR Evidence Synthesis Group @Complex Review Support Unit; Health Economics and Health Technology Assessment (HEHTA), School of Health and Wellbeing, University of Glasgow, Glasgow, UK; NIHR Evidence Synthesis Group @Complex Review Support Unit; Department of Population Health Sciences, University of Leicester, Leicester, UK; NIHR Evidence Synthesis Group @Complex Review Support Unit; Department of Population Health Sciences, University of Leicester, Leicester, UK; NIHR Evidence Synthesis Group @Complex Review Support Unit; Health Economics and Health Technology Assessment (HEHTA), School of Health and Wellbeing, University of Glasgow, Glasgow, UK; NIHR Evidence Synthesis Group @Complex Review Support Unit; School of Cardiovascular and Medical Sciences, University of Glasgow, Glasgow, UK

**Keywords:** ageing, evidence, meta-analysis, methods, systematic review, older people

## Abstract

Evidence synthesis, embedded within a systematic review of the literature, is a well-established approach for collating and combining all the relevant information on a particular research question. A robust synthesis can establish the evidence base, which underpins best practice guidance. Such endeavours are frequently used by policymakers and practitioners to inform their decision making. Traditionally, an evidence synthesis of interventions consisted of a meta-analysis of quantitative data comparing two treatment alternatives addressing a specific and focussed clinical question. However, as the methods in the field have evolved, especially in response to the increasingly complex healthcare questions, more advanced evidence synthesis techniques have been developed. These can deal with extended data structures considering more than two treatment alternatives (network meta-analysis) and complex multicomponent interventions. The array of questions capable of being answered has also increased with specific approaches being developed for different evidence types including diagnostic, prognostic and qualitative data. Furthermore, driven by a desire for increasingly up-to-date evidence summaries, living systematic reviews have emerged. All of these methods can potentially have a role in informing older adult healthcare decisions. The aim of this review is to increase awareness and uptake of the increasingly comprehensive array of newer synthesis methods available and highlight their utility for answering clinically relevant questions in the context of older adult research, giving examples of where such techniques have already been effectively applied within the field. Their strengths and limitations are discussed, and we suggest user-friendly software options to implement the methods described.

## Key Points

- To accompany the *Age and Ageing* journal special collection on Evidence Synthesis in Older Adults.
- Systematically collating and summarising published research is a key component of evidence-based healthcare.
- As clinical questions are increasingly complex, approaches to synthesis of the evidence are now necessarily more sophisticated to answer the most relevant questions in contemporary older adult care.
- Methods to allow direct and indirect comparisons and synthesis of multiple interventions, qualitative, test accuracy and prognosis research have all been described and have been applied in areas relevant to older adult care.
- There is much scope for further uptake and application of newer synthesis methods to older adult care research and user-friendly software options are becoming available to make such methods more accessible to the research community at large.

## New horizons in evidence synthesis in geriatric medicine

It is often said that the problem with taking an evidence-based approach to the care of older adults is the lack of any primary evidence. Whilst it is true that, relative to other clinical areas, geriatric medicine suffers from a lack of primary research, [1] it is not true that there is no evidence relevant to older adults. Bringing together the available research on a particular topic can often give answers that are greater than the sum of the constituent parts.

**Figure 1:**
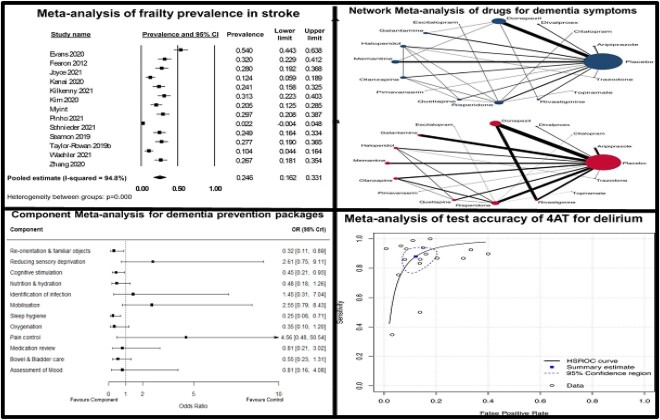
Summary data outputs from four differing evidence synthesis methods. Clockwise from top left: forest plot describing summary estimate of prevalence of frailty in acute stroke [53], NMA of pharmacological interventions for neuropsychiatric symptoms in dementia [54], DTA of the 4AT delirium screening tool [55], effectiveness of individual components in a multicomponent intervention for delirium prevention [21].

**Figure 2:**
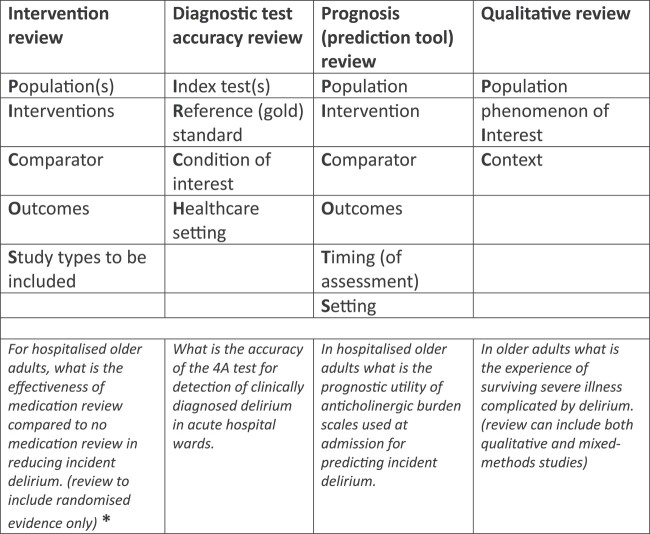
Differing methods suitable for evidence synthesis and the differing approaches to formulating the review question. * See Figure 3 for more details of how other questions and more complex data structures can be examined.

The science around this process of evidence synthesis is rapidly evolving, with new methods and approaches to allow the incorporation of many differing evidence types. The standard paradigm of a comprehensive review of the literature, followed by a meta-analysis of quantitative data comparing two study arms, remains a valid approach, but may not always be suited to the increasingly complex healthcare issues that face clinicians and policymakers. In this review, we will consider some of the newer approaches that have been described in the field of evidence synthesis, and how they could be applied to older adult research (Figures 1 and 2). The methods we consider here are not an exhaustive list, and discussions of other methods including synthesis without meta-analysis, individual participant data synthesis and synthesis of real-world data are available elsewhere [2–4]. We will also not discuss the fundamentals of evidence synthesis methods, but for the reader wishing a review of this topic there have been recent relevant papers [5].

## ‘Old horizons’ in evidence synthesis

The differing evidence synthesis methods available, all tend to have a similar basic structure: systematic search of the literature for relevant papers, extraction of data according to a pre-specified protocol, assessment of quality of the collated evidence, and finally synthesis of the data to create a summary of the body of research. Although still considered a relatively new approach in research, this concept of collating evidence and creating summary analyses has been understood for at least 100 years [6], indeed systematic review, and meta-analysis as we know it, is probably now in its fourth decade [7]. There is no doubt that for providing unbiased estimates of treatment effect, the systematic review paradigm is a major improvement on the traditional non-systematic literature review. However, the growth of the systematic review has not come without criticism. Some of the early adopters and advocates of this facet of evidence-based medicine are now vocal in their opposition to the perceived ‘explosion’ of reviews. It has been suggested that for many topics there are now more reviews than there are primary research studies [8].

Whilst, quite rightly, the increasing prevalence of duplicate, poorly reported or methodologically dubious systematic reviews has been criticised, this does not imply that the process is fundamentally flawed. Instead, it should be a call to action for researchers, funders and journal editors to ensure that best practice is followed. In this regard, particular attention should be given to pre-registering a protocol on a publicly accessible database such as the prospero resource, ensuring adequate expertise is used in designing the search strategy/running the analyses, and following appropriate guidelines when reporting [9].

## Network meta-analysis

Historically, reviews would compare and collate (quantitatively with a meta-analysis) trials of a single intervention versus a control, when in practise, there often exists a plethora of different interventions for a given condition. Therefore, it is increasingly important for clinicians, academics and decision-makers to understand the comparative effectiveness of all the available options. Suboptimal methods to address this include comparing multiple treatments in a simple pairwise meta-analysis or performing multiple pairwise meta-analyses. The former is not always practical or useful and can lead to the lumping of many active interventions, whereas the latter is difficult to interpret [10].

Network meta-analysis (NMA), or mixed treatment comparisons, is an extension of pairwise meta-analysis that allows comparison of more than two interventions simultaneously. Like pairwise meta-analysis, NMA can evaluate various outcome types, such as dichotomous, continuous and time-to-event data, using the suitable scale for each type [10, 11]. NMA uses both direct (head-to-head) and indirect (via a reference treatment) comparisons to provide relative effect estimates. It can also incorporate multiarmed trials (more than two arms) whilst avoiding double counting of participants [11]. Because of this ability to analyse indirect effect estimates, NMA becomes a useful tool where a direct comparison of treatments is not available or possible.

For example, an NMA for interventions to prevent falls in older adults compared 39 interventions with usual care and estimated the comparative effectiveness of all interventions, even when trials with head-to-head comparisons of some treatments were not available [12]. Although statistically more complex, it can be seen how the resulting NMA makes better use of available data and is more useful for making choices about the best intervention. Another advantage of NMA is its ability to rank the available treatment options in accordance with their effectiveness. An NMA for various exercise interventions for people with Parkinson’s disease found that interventions like dance and aqua-based training were ranked highest for effectiveness in improving motor signs [13].

There are situations where the interventions cannot be compared either directly or indirectly leading to what is called a disconnected network [14]. There are several proposed approaches to deal with disconnected networks: utilising common intervention components (see below), [15] using population adjustment methods and propensity score matching methods when partial or full individual participant data are available, [16, 17] or by using non-randomised evidence [17].

User-friendly, and free to access, statistical packages are available and allow for users to perform NMA without the need for specialist software or code, for example, the Meta-Insight app [18]. This approach democratises NMA but the conduct of the analyses and the interpretation of the data still requires specialist expertise.

### Component NMA

**Figure 3:**
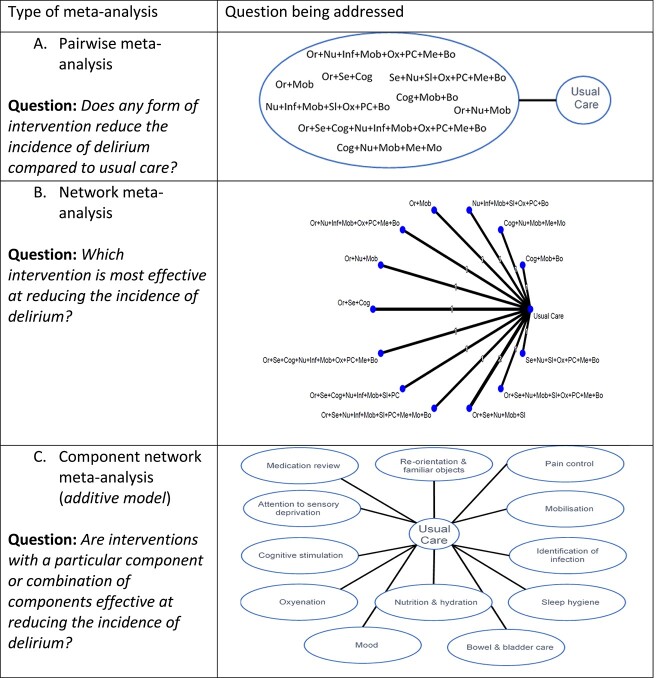
(A, B, C) Examples of the different types of meta-analysis models and the questions they address when applied to the example of delirium. Or = re-orientation and familiar objects, Se = attention to sensory deprivation, Cog = cognitive stimulation, Nu = nutrition and hydration, Inf = identification of infection, Mob = mobilisation, Sl = sleep hygiene, Ox = oxygenation, PC = pain control, Me = medication review, Mo = mood, Bo = bowel and bladder care.

In geriatric medicine, complex or multicomponent interventions are increasingly prevalent. Historically, a pairwise meta-analysis was used where all the interventions were ‘lumped’ together under a common heading of multicomponent interventions. This aggregate approach allowed for summary analyses but with no consideration of the individual factors included in the multicomponent interventions. For example, a 2016 pairwise meta-analysis on delirium prevention combined all non-pharmacological and pharmacological approaches into a single ‘intervention node’. At best, this answers the question ‘does any form of intervention reduce the incidence of delirium compared to usual care?’, but is not informative about the nature of the optimal intervention and thus is of very limited use for clinical decision-making (Figure 3A) [19]. Indeed, if there are harmful and beneficial interventions lumped together, an overall negative result may mask that some interventions were effective.

Whilst NMA can answer questions such as ‘which intervention is most effective at reducing the incidence of delirium?’ (Figure 3B), these interventions often consist of multiple components. Randomised controlled trials (RCTs) of multicomponent interventions may evaluate treatment strategies that have at least some components in common with the strategies evaluated in other trials. For example, available trials of non-pharmacological interventions for preventing delirium in hospitalised non-intensive care unit patients may all include ‘mobilisation’ but some may also include ‘re-orientation and familiar objects’, whereas others include ‘nutrition’ (Figure 1, top right panel). Synthesising multicomponent interventions using NMA can result in a network with many intervention nodes but few trials connecting the intervention nodes to anything other than ‘usual care’ (and networks may sometimes be disconnected altogether). This can lead to fitting a model with many parameters but few trials contributing to their estimation, resulting in large amounts of uncertainty surrounding the intervention effects and giving little insight into the relative contribution of individual components or how they interact with each other.

An alternative approach is component network meta-analysis (CNMA) where the model estimates the effect of each component (Figure 3C) and can predict the effectiveness for any and every combination of components including combinations not previously included in trials [20]. Therefore, CNMA has the ability to answer questions such as ‘are interventions with a particular component or combination of components effective?’ and ‘what are the most effective packages of care?’. For example, in the CNMA of non-pharmacological interventions for preventing delirium, it is possible to predict the effectiveness of the combination ‘orientation, mobilisation and pain control’, even if no primary trial considered this combination [21].

CNMA was first proposed in 2009 and through a series of network meta-regression models can estimate the effect of each component and combinations of components in a network [22]. The simplest model is the additive effects model in which the effect of a combination of components is assumed equal to the sum of the effects of the individual components. Synergistic or antagonistic effects of components given in combination can be accounted for by extending the additive effects model to allow for interactions between pairs of components. In recent years, there has been an increase in the use and methodological development of this CNMA modelling approach, for evaluating both public health interventions and for combinations of drug treatment [23–25].

CNMA can be conducted using a frequentist approach in R or a Bayesian approach in WinBUGS. The R packages netmeta [26] and viscomp [27] offer graphical options for visualising the data structure and results.

## Qualitative approaches

The value of qualitative research in health care of older adults has been recognised for many years [28]. Qualitative evidence synthesis (QES) is an umbrella term used to describe more than 30 different methods, developed over the last four decades against a backdrop of growing demand for review evidence that extends beyond ‘what works’ and can shed light on the reasons ‘why’ interventions work (or fail to work) [29]. QES approaches have traditionally been distinguished into integrative approaches that seek to summarise or ‘aggregate’ findings from individual studies (e.g. narrative synthesis, framework synthesis and thematic synthesis without theory development), and interpretive approaches that use original findings as a basis for generating new interpretations and conceptual understandings, e.g. meta-ethnography, grounded theory and thematic synthesis involving theory development [30]. Selection of the most appropriate QES method for a particular review is usually based on a number of considerations, including, but not limited to, the type of review question (i.e. integrative approaches tend to address ‘fixed’ or pre-determined questions, whereas interpretive methods lend themselves well to emergent questions), the target audience and intended purpose of the review (i.e. outputs from integrative methods are in general more straight-forward and thus might be more appealing to certain audiences, such as policy-makers, compared with the outputs from interpretive methods that are more complex and conceptual) and the type of data identified (i.e. when primary studies are mostly descriptive in nature and lack conceptual depth, using integrative methods might be the only option) [31].

Despite notable differences, what all QES methods have in common is a shared goal of systematically bringing together the findings from diverse qualitative studies, in order to make them more easily accessible and usable for healthcare policy and practice. In the same way that the pooling of quantitative studies in meta-analysis is driven by the promise of achieving greater statistical power and more accurate results, synthesising qualitative studies is motivated by the prospect of enhancing explanatory power and producing something that is of greater utility than considering each of the individual studies [32]. For example, a recent meta-ethnography of older adults’ experiences of physical activity sought to determine what available qualitative research can contribute to explaining current failures to increase population levels of activity in this age group [33]. Drawing on 37 qualitative studies and two systematic reviews, the authors were able to generate a theory of physical activity in the context of older adults, where transition to older age can challenge individuals’ sense of self, and physical activity can play an important role in regaining feelings of purpose, being needed in collective group activity, and creating habitual routine and structure to the day. The review was then able to give practical applications for future trials, suggesting that rather than emphasising the health benefits of physical activity, intervention developers need to reframe their approach to consider the wider set of goals and aspirations that are of importance to older adults, such as having a purposeful and fulfilling life.

## Diagnostic test accuracy

Important issues in older adult care are not only centred around whether interventions work, but also around diagnosis and assessment of health and disease. Questions such as, ‘what is the best tool to screen for frailty’ or ‘does this person have dementia’ lend themselves to the diagnostic test accuracy (DTA) approach [34]. Definitive assessment of test accuracy usually requires large populations, but primary studies in DTA are often modest in size. Systematic review and meta-analysis of DTA data can help provide increased precision in estimates of accuracy and can be used to consider how accuracy can vary according to context.

Whilst the basic approach is similar to other review types, there are additional complexities with systematic review and meta-analysis in the DTA space. The underlying structure of the review question moves from the standard ‘PICO’ approach (population, intervention, control and outcome) to a consideration of the index test(s), reference (or gold) standard, condition of interest and healthcare setting [35]. Accuracy is traditionally described using complementary metrics of sensitivity and specificity, and so the meta-analytical techniques need to capture both these measures. To allow for ease of interpretation, the data may be presented in receiver operating characteristic space with visualisation of summary estimates and corresponding confidence and prediction intervals [36].

Most DTA meta-analysis compare one test in isolation, for example, reviews of cognitive tests such as the Addenbrookes Cognitive Examination [37] or the Montreal Cognitive Assessment [38]. However, in practice, the question of interest is not usually how accurate is this test, but rather which of the available tests is most accurate. Drawing upon a similar theory to NMA, approaches that allow direct and indirect comparison and ranking of multiple tests have been described and one has been applied to allow comparison of multiple cognitive screening tools [39].

Another common situation in clinical practice is where the reference or ‘gold’ standard is a clinical diagnosis. For example, tests of delirium screening tools where the reference standard is expert clinical diagnosis. However, even when using standardised approaches clinical diagnosis often has associated uncertainty or poor reliability, the so called ‘imperfect’ reference standard. Again, recent techniques have been described to allow incorporation of this uncertainty into the summary estimates of accuracy and even estimate the accuracy of the imperfect reference standard [40].

## Prognosis research

Clinical prognostic research attempts to describe the natural history of a condition or identify those variables that can best predict outcomes. Prognosis research can take various approaches including fundamental prognosis (natural history of a condition), assessing prognostic factors or combining these factors into multi-item prediction tools [41]. Regardless of the approach, the prognosis method is essential for health care planning, policy and in the identification of novel targets for interventional research.

A typical example of prognostic factor research in the older adult population would be investigating the association of anticholinergic medications with subsequent dementia [42]. A related prediction tool research question would be whether the assessment of various factors in midlife, when combined in a model or prediction tool, can predict subsequent cognitive decline [43]. In both examples, there have been many primary research papers published, although most are modest in size or restricted to specific populations. The use of evidence synthesis methods allows for a summary estimate with greater precision and can be used to explore the heterogeneity between populations.

Prognostic research can be prospective in nature but is more often conducted using data that was obtained for an alternative purpose (e.g. secondary analysis of data obtained from a randomised controlled trial or cohort). Whilst this approach allows for quick and efficient use of resources with minimal costs, the reliance on pre-existing data limits the control investigators have to design their study according to the specifications required to thoroughly investigate the relationship between a variable and an outcome. It is frequently the case that the variables of greatest interest to a prognostic study are measured sub-optimally or not measured at all within the data sets available. These issues can be magnified when trying to combine data sets in an evidence synthesis.

Related to this, there are often significant issues regarding the reporting of prognostic studies. Considering our example of anticholinergic exposure and dementia, key details, such as how long a person has been exposed to anticholinergic drugs, the dosage of drug or the type of dementia they subsequently develop, are frequently not available [42]. Again, whilst this is a problem for interpretation of the primary research, the issues become magnified when trying to combine papers in a meta-analysis. This highlights the importance of using reporting guidance in papers describing prognosis research [44].

These factors can result in significant heterogeneity between conceptually similar studies. For instance, in anticholinergic burden research, heterogeneity is evident in the cognitive tests employed to measure outcomes, time-points at which outcomes are evaluated and the variables controlled for in statistical analyses. This culminates in major issues with attempts to synthesise evidence through meta-analysis. These issues can be alleviated in part by standardising outcomes (e.g. using *z*-scores) where possible, grouping studies according to variable measurement methods and ensuring that only studies that controlled for ‘core’ variables (e.g. age, sex and other variables that have been consistently associated with the outcome of interest) are included in meta-analysis [45].

Lastly, the retrospective nature of prognostic research often means that no protocol is registered prior to a study’s conduct and completion. This increases the potential risk for publication bias and prohibits the ability to scrutinise ‘planned’ analyses against ‘reported’ analyses. Pre-registration of statistical analysis plans on publicly accessible databases would help diminish the effect of this widespread issue.

## Living systematic reviews

For healthcare decisions, policy and especially for guidelines it is best practice to consider all the relevant evidence that is available. However, whilst the methods for conducting a systematic review are well established, they are often labour and resource intensive, leading to delays in incorporating new evidence [46]. As a result, traditional systematic reviews can easily become outdated and risk offering inaccurate or misleading summary evidence. If this evidence is then used in a clinical practice guideline, the recommendations may not represent evidence-based care. In response, a new approach to updating reviews has recently been proposed—living systematic reviews (LSRs), where the continually updated review incorporates new evidence as it becomes available [47].

**Figure 4:**
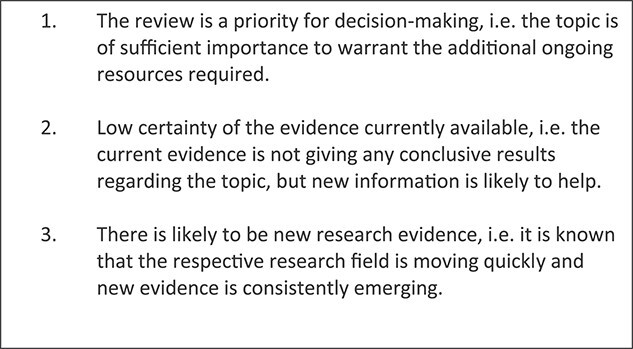
Factors to consider when approaching a possible ‘living’ systematic review.

Whilst LSRs improve the timeliness and relevance of reviews, the ‘living’ elements can be resource-intensive and the practicalities of the method are still being developed [48]. Thus at present, the benefits of the LSR approach may not always outweigh the costs and resources required. Elliott *et al*. [47] suggest three criteria to help researchers decide when an LSR is appropriate (Figure 4).

Within the field of ageing, there are various research areas where these criteria of high-volume evidence and clinical uncertainty could apply and where an LSR could be feasible, including interventions for sarcopenia, new pharmacological treatments for dementia and management of frailty. For some areas, LSRs already exist; for example, the effectiveness and safety of treatments to prevent fractures in people with osteoporosis or living guidelines on stroke care [48, 49]. The process not only allows for syntheses of available evidence but can also direct future primary research. In the first update of the osteoporosis LSR, the authors made conclusions regarding treatment effects for postmenopausal females, but noted that more studies were needed on sequential therapy and for males [49]. As the review is living, researchers can incorporate such evidence as it emerges. LSRs became of particular importance during the COVID-19 pandemic, and applications relevant to older adults were seen. For example, following reports that the greatest mortality and morbidity risks were in older adults living with frailty and other vulnerable groups, a ‘rapid’ LSR was conducted to provide updates on treatment and rehabilitation needs in this group [50].

## Newest horizons in evidence synthesis

Whilst the methods described here are relatively novel, and many are still being developed and refined, even newer developments for evidence synthesis are on the horizon. The increasing power of artificial intelligence will allow for automation of many aspects of the review and analysis process, reducing the time, staff and economic burden of review production [51]. Of particular relevance to older adults, and other populations, are methods to allow incorporation of covariates into meta-analyses, thus reducing biases because of differences between studies and allowing exploration of how effectiveness varies by patient characteristics [52]. The increasing availability of research data set repositories will facilitate greater application of individual participant level data analyses, and approaches for combining participant-level and trial-level data are available. The format for presenting evidence synthesis is also likely to change, moving from traditional print-style static manuscripts to interactive resources that allow readers to assess subgroups and covariates.

## Conclusion

As healthcare and healthcare questions have become more complicated, techniques for summarising these data in a way that is useful to clinicians, academics and policymakers have also had to become more sophisticated. The systematic review and meta-analytical paradigm are no longer confined to analysis of simple placebo controlled RCTs, and can now be applied to primary complex interventions, diagnostic, prognostic and qualitative research. The opportunities are varied and exciting, but for both the evidence synthesis and the primary research that forms the basis for these reviews, there is still the requirement for engagement between clinicians and methodologists and the need to follow best practice in conduct and reporting.
